# Three-Color Chromosome Painting as Seen through the Eyes of mFISH: Another Look at Radiation-Induced Exchanges and Their Conversion to Whole-Genome Equivalency

**DOI:** 10.3389/fonc.2016.00052

**Published:** 2016-03-15

**Authors:** Bradford D. Loucas, Igor Shuryak, Michael N. Cornforth

**Affiliations:** ^1^Department of Radiation Oncology, University of Texas Medical Branch, Galveston, TX, USA; ^2^Center for Radiological Research, Columbia University, New York, NY, USA

**Keywords:** chromosome painting, mFISH, radiation biomarkers

## Abstract

Whole-chromosome painting (WCP) typically involves the fluorescent staining of a small number of chromosomes. Consequently, it is capable of detecting only a fraction of exchanges that occur among the full complement of chromosomes in a genome. Mathematical corrections are commonly applied to WCP data in order to extrapolate the frequency of exchanges occurring in the entire genome [whole-genome equivalency (WGE)]. However, the reliability of WCP to WGE extrapolations depends on underlying assumptions whose conditions are seldom met in actual experimental situations, in particular the presumed absence of complex exchanges. Using multi-fluor fluorescence in situ hybridization (mFISH), we analyzed the induction of simple exchanges produced by graded doses of ^137^Cs gamma rays (0–4 Gy), and also 1.1 GeV ^56^Fe ions (0–1.5 Gy). In order to represent cytogenetic damage *as it would have appeared to the observer* following standard three-color WCP, all mFISH information pertaining to exchanges that did not specifically involve chromosomes 1, 2, or 4 was ignored. This allowed us to reconstruct dose–responses for three-color *apparently simple* (AS) exchanges. Using extrapolation methods similar to those derived elsewhere, these were expressed in terms of WGE for comparison to mFISH data. Based on AS events, the extrapolated frequencies systematically overestimated those actually observed by mFISH. For gamma rays, these errors were practically independent of dose. When constrained to a relatively narrow range of doses, the WGE corrections applied to both ^56^Fe and gamma rays predicted genome-equivalent damage with a level of accuracy likely sufficient for most applications. However, the apparent accuracy associated with WCP to WGE corrections is both fortuitous and misleading. This is because (in normal practice) such corrections can only be applied to AS exchanges, which are known to include complex aberrations in the form of pseudosimple exchanges. When WCP to WGE corrections are applied to *true simple exchanges*, the results are less than satisfactory, leading to extrapolated values that *underestimate* the true WGE response by unacceptably large margins. Likely explanations for these results are discussed, as well as their implications for radiation protection. Thus, in seeming contradiction to notion that complex aberrations be avoided altogether in WGE corrections – and in violation of assumptions upon which these corrections are based – their inadvertent inclusion in three-color WCP data is *actually required* in order for them to yield even marginally acceptable results.

## Introduction

Whole-chromosome painting (WCP) involves the labeling of a few select chromosomes of the genome, thereby producing discrete changes in fluorescent color patterns that accompany the junctions of exchange breakpoints. These include junctions between the painted and unpainted chromosomes, and between the painted chromosomes themselves.

Whole-chromosome painting data can be extrapolated in order to approximate the total number of exchanges that *would have been detected* if all homologous chromosome pairs *would have been painted* a unique color, as in the combinatorial painting technologies of multi-fluor fluorescence *in situ* hybridization (mFISH) (1) or spectral karyotyping (SKY) (2). Converting WCP data to that of whole-genome equivalency (WGE) provided by mFISH or SKY makes use of relationships similar to that developed by Lucas and colleagues (3). These consider exchanges between painted and unpainted (counterstained) chromosomes, adjusting for unseen exchanges presumed to have occurred between unpainted chromosomes. After three-color WCP was introduced, subsequent refinements were made to accommodate exchanges occurring among the individually painted chromosomes as well (4, 5).

There are two central assumptions common to these mathematical extrapolations. First, that exchange breakpoints are produced *randomly* throughout the genome, in direct proportion to the size of chromosomes participating in an exchange. Second, these corrections (extrapolations) are derived solely in consideration of simple reciprocal interchange events (dicentrics and translocations). Complex exchanges, which involve rejoining among multiple chromosomes, are ignored. For that reason, corrections are usually restricted to data associated with low to moderate doses of X- or gamma rays, where the incidence of complex exchanges is assumed to be minimal. Earlier work provided support for the soundness of the basic approach and whole-genome corrections soon became routinely applied to WCP (3, 6–8) data. However, papers began to appear shortly thereafter questioning the first of the aforementioned assumptions (9–11).

More recently, and with basic intent similar to ours, Braselmann and colleagues compared genome-corrected three-color WCP data with experimental data derived independently using mFISH and SKY (4). When applied to three-color WCP data, they found that modification to the original Lucas formula (3) produced results comparable to that of mFISH or SKY. Attached to this conclusion, however, was a cautionary note about the influence of pseudosimple exchanges – aberrations that appear to be simple pairwise interchanges by WCP, but that are actually complex, involving three or more exchange breakpoints distributed among multiple chromosomes (12–14).

In this paper, we reconsider the issue in detail by comparing 24-color mFISH data to 3-color data retrospectively extracted from mFISH images. This method was used to assess the accuracy with which a commonly used mathematical formalism can be applied 3-color WCP data in order to extrapolate full 24-color genome equivalency for simple chromosome interchanges. It involves experimental conditions under which WCP extrapolations are ostensibly valid, such as low to moderate doses of gamma rays. Unlike previous reports, however, it also includes situations where the validity of such extrapolation is dubious: higher doses of gamma rays and exposure to heavy ions, both of which are well known to favor the production of complex exchanges (5, 15–22).

## Materials and Methods

### Irradiations and Culture Conditions

Methods pertaining to the exposure of lymphocytes to gamma rays have been detailed elsewhere (22, 23). Whole venous blood from two healthy consenting male volunteers was exposed to graded doses of ^137^Cs γ-rays at a rate of 1.3 Gy/min using a J.L. Shepherd Mark I cesium irradiator located at the University of Texas Medical Branch (UTMB), following procedures approved by UTMB’s Institutional Review Board (IRB). 0.4 ml aliquots of blood were cultured in RPMI-1640 (Gibco) medium containing 0.1 ml phytothemagglutinin (PHA; Murix, Dartford, UK) and supplemented with 15% fetal bovine serum. Colcemid (GIBCO), to a final concentration of 0.1 μg/ml, was added 45 h later, and cultures were harvested for metaphase analysis at 48 h.

Heavy ion irradiations took place at Brookhaven National Laboratory (BNL; Upton, NY, USA) within the NASA Space Radiation Laboratory (NSRL). Procedures followed those of BNL’s IRB. Whole blood was suspended in RPMI-1640 medium, supplemented with 20% fetal bovine serum. From this suspension, approximately 2 × 10^6^ cells were loaded into custom-made Lucite holders and irradiated at room temperature with graded doses of 1.1 GeV/amu ^56^Fe ions. The dose average LET of this beam was 147 keV/μm. Immediately after exposure, lymphocytes were aspirated from the holder and transferred into 25 cm^2^ tissue culture flasks containing 10 ml of RPMI-1640 medium supplemented with 1% phytohemagglutinin (PHA; Gibco). Cultures were incubated at 37°C for 46 h before Colcemid (Gibco) was added (0.2 μg/ml final concentration) 2 h prior to the harvest of mitotic cells. Calyculin-A (50 nM final concentration) was added to Colcemid-blocked cultures to induce premature chromosome condensation (PCC) in G_2_-phase cells (24). As a result, mitotic figures contained a mixture of metaphase chromosomes and G_2_-phase PCC. Cells were fixed in a 3:1 mixture of methanol to acetic acid and transported to the University of Texas Medical Branch at Galveston for further processing and subsequent analysis.

### mFISH Hybridization and Image Capture

Following fixation in methanol/acetic acid, lymphocytes were spread onto glass microscope slides by standard cytogenetic procedures. Slides were then treated with acetone, RNase A, and proteinase K before another fixation in 3.7% formaldehyde. Slides were dehydrated through an ethanol series (70, 85, and 100%) and air dried. In order to denature chromosomal DNA, they were next incubated in 70% formamide (72°C) in 2× SSC (0.3 M NaCl, 0.03 M sodium citrate) for 2 min. After dehydration through another ethanol series, 10 μl of denatured (10 min at 72°C) SpectraVision 24-color mFISH Assay probe (Vysis) was applied to each slide. Slides were covered with a 22 mm × 22 mm glass cover slip, sealed into position with rubber cement. Samples were allowed to hybridize for 48 h in a 37°C incubator. Following hybridization, cover slips were removed and the slides were washed for 2 min in 0.4× SSC containing IGEPAL (0.3%) non-ionic detergent at 72°C. This was followed by a 30-s wash in 2× SSC (0.1% IGEPAL) at room temperature.

Prior to image capture, 15 μl of DAPI (0.14 μg/ml) dissolved in anti-fade mounting medium (Vectashield; Vector Laboratories) was applied to each slide and covered with a 24 mm × 40 mm cover slip. Images of chromosome spreads were captured using a Zeiss Axiophot epifluorescence microscope interfaced with a SensSys black-and-white CCD camera. Karyotypes were constructed from good-quality chromosome spreads using PowerGene image analysis software (23).

### 24-Color Analysis

We conducted a retrospective examination of a large 24-color mFISH data base that contained detailed information on aberrations produced in human cells by graded doses radiations of different ionization densities (22, 23). Metaphase cells were analyzed by procedures previously established (23). Briefly, mPAINT descriptors were assigned to chromosomes involved in each rearrangement. Next, each rearrangement was brought to “pattern closure” by grouping elements in the most conservative way possible, minimizing the number of breakpoints required to reconstruct the exchange (25). Reciprocal pairwise rejoinings between one chromosome (rings and interstitial deletions) or two chromosomes (translocations and dicentrics) were scored as simple exchanges. Exchanges involving three or more breakpoints were regarded as complex. This classification was also applied to incomplete exchanges where one or more elements failed to rejoin, as well as the so-called “one-way” exchanges where one or more translocated segments appeared to be missing, presumably because they were too small to be resolved by chromosome painting. The large majority of one-way staining patterns are known to be complete exchanges (26). And since we lacked the ability to simultaneously visualize telomere signals in mFISH preparations, such rearrangements were treated as being complete for the purpose of achieving pattern closure.

### Retrospective Three-Color Analysis

We focused on chromosomes 1, 2, and 4, since this represents one of the more commonly used three-color painting schemes. On a cell-by-cell basis, we stripped from the full 24-color mFISH profile all information concerning exchanges *except* that pertaining to the three painted chromosomes. In other words, from a full 24-color karyotype, we imagined what the microscopist *would have observed* if, instead of mFISH, three-color WCP had been applied to the samples. From this information, we used a mathematical correction of the form described by Braselmann et al. (4) to scale WCP data back to full genome equivalency originally provided by mFISH. The correction we used applies only to simple reciprocal interchanges involving exactly two chromosomes (translocations and dicentrics). Neither mFISH nor WCP analysis specifically considered intrachanges: rings, interstitial deletions, inversions; nor were terminal deletions considered. To be clear then, the term “exchange” (as used hereafter) refers only to interchanges. One-way exchanges were handled in a manner similar to that for mFISH.

### Extraction of Three-Color Data from mFISH Images

**Figure 1:**
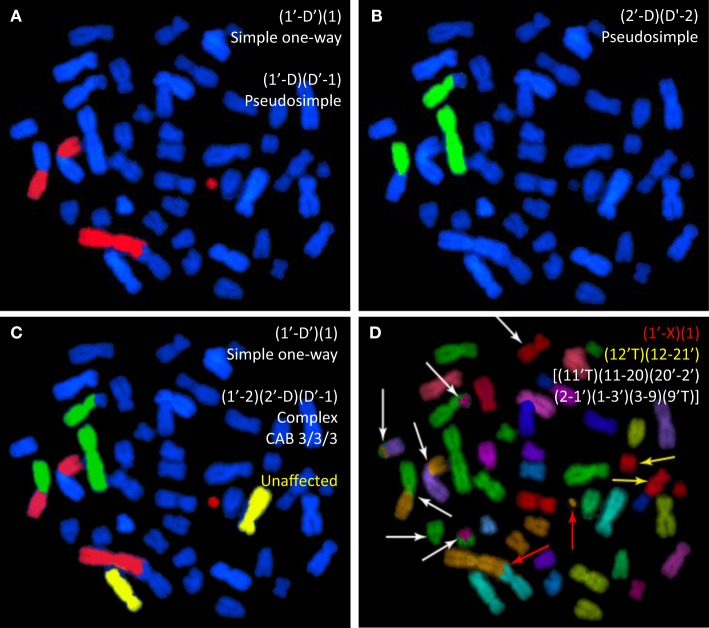
**Whole-chromosome paints applied to a human metaphase spread from a cell previously exposed to gamma rays**. The same spread is shown following WCP applied to chromosomes 1 and 2 [**(A,B)**, respectively]. **(C)** The same spread following simultaneous painting with the same two probes. In **(D)**, mFISH reveals the full extent of exchange complexity. mPAINT nomenclature is used to describe the various visible rearrangements (25).

Figure 1 depicts the process used in rendering 24-color mFISH images in order to produce 3-color WCP data. It is also meant to illustrate some of the problems inherent to WCP for aberration analysis. The figure shows various staining protocols applied to a metaphase cell that had previously been exposed in G_0_ phase to 4 Gy of ^137^Cs gamma rays. The cell is replete with various chromosome rearrangements whose complexity becomes increasingly apparent as different chromosomes are painted. Panels A, B, and C derive from an mFISH image that was rendered to exclude painting information from all chromosomes *except* chromosomes 1, 2, and 1 + 2 + 4, respectively.

Figure 1A is of a cell probed for chromosome 1 that contains an apparently simple (AS) translocation between chromosome 1 and an anonymous blue (DAPI-counterstained) chromosome. The cell also contains an AS dicentric involving the other homolog of chromosome 1. [In this case, the accompanying compound acentric fragment shows a “one-way” staining pattern, and is therefore assumed joined with a submicroscopic counterstained segment (26–29)]. Figure 1B shows the same cell, as it would appear if probed for chromosome 2 instead. Here, an AS translocation has occurred. Figure 1C simulates the three-color painting patterns of the same cell that derive from mFISH data, rendered so as to include data for chromosomes 1, 2, and 4 simultaneously. The full extent of complexity is revealed by mFISH in Figure 1D. Actually, the cell in question is shown to harbor three rearrangements. It contains a simple dicentric between chromosome 1 and the X (red arrows). This exchange would be correctly identified given the staining patterns shown in Figures 1A,C. Judging by staining patterns of Figure 1A, it also contains a simple translocation involving the homologous chromosome 1. In reality, the exchange is pseudosimple. mFISH reveals the chromosome to be part of a large complex exchange involving five other chromosomes marked by white arrows. Likewise, the AS translocation involving chromosome 2 is also pseudosimple, since mFISH shows it to be part of the same large complex exchange (white arrows).

The three-color rendering shown in Figures 1C represents the type of WCP data to which the CF corrections of equation (8) (shown below) were applied in order to calculate WGE. In this particular cell, three-color painting was able to detect the occurrence of the complex exchange. However, from the three-color staining pattern alone, one may conclude only that the complex involved a minimum of three chromosomes: 1, 2, and an anonymous third DAPI-stained chromosome, when six chromosomes were actually involved (Figure 1D). In fact, there are many instances where three-color painting fails to detect the occurrence of complex exchanges altogether. The misidentification of complex exchanges as being simple is of concern to mathematical extrapolations applied to three-color data, because it violates a central assumption that only simple exchanges be considered, a point made repeatedly in this paper.

### Extrapolation to Whole-Genome Equivalency

Over the years, various modifications to the original Lucas formula (3) have been used to estimate the fraction of total interchanges visible by WCP. The extrapolation we used follows closely that of Braselmann and colleagues (4). It considers exchanges between painted and unpainted (counterstained) chromosomes, as well as exchanges taking place among the uniquely painted chromosomes. It also makes provisions for the fact that dicentrics involving homologous chromosomes are detectable by mFISH, whereas translocations are not. Values for the genomic content of chromosomes used in the following derivation are from Mendelsohn et al. (30) as cited by Morton (31). Its derivation, as applied to our particular experimental system, is as follows.

Let *f_p_* represent the fractional sum of the genome covered by the individual chromosomes 1, 2, and 4, where *f_1_* = 0.0821; *f_2_* = 0.0804; *f_4_* = 0.0635

(1) $$f_{p}=\left(f_{1}+f_{2}+f_{4}\right)=0.226.$$

The unpainted (DAPI-counterstained) fraction then becomes

(2) $$\left(1-f_{p}\right)=0.774$$

For WCP, the frequency of visible interchanges in the genome that can occur between painted and unpainted chromosomes (*F_P_*) is given by the cross product of the binomial expansion (*p* + *q*)^2^ = *p*^2^ + 2*pq* + *q*^2^ – namely 2pq – where *p* = (*f_p_) and q* = 1−(*f_p_)*. Substituting values in Eq. (2) gives the following expression.

(3) $$F_{P}=2\mathrm{pq}=2f_{p}\left(1-f_{p}\right)=0.350$$

If, as is the case here, the individual painted chromosomes can be distinguished from one another, then Eq. (3) can be expanded to include exchanges that now become visible among the three possible pairs of uniquely colored chromosomes (4, 32).

(4) $$F_{P}=2\left[f_{p}\left(1-f_{p}\right)+f_{1}f_{2}+f_{1}f_{4}+f_{2}f_{4}\right]=0.384$$

Thus, three-color WCP is theoretically capable of detecting 38.4% of the interchanges occurring throughout the whole genome. However, in the context of this paper, three-color WCP frequencies are to be compared to those detected by mFISH and it should be recognized that the latter is not capable of detecting all interchanges. The frequency of all mFISH-detectable interchanges (*F_mFISH_*), including translocations and dicentrics, is proportional to the sum of all products (*f_i_) x (f_j_)* representing the fractional DNA content of chromosomes *i* and *j*. But because mFISH cannot reliably detect events that occur between homologous chromosomes, an additional stipulation is that *i ≠ j*. For a human karyotype containing 23 individually identifiable types of chromosomes, this can be represented by the following expression (4).

(5) $$F_{\mathrm{mFISH}}=1-\sum_{i=1}^{23}f_{i}^{2}=0.948$$

The numerical value resulting from Eq. (5) is essentially a constant for a given diploid species. We note that our calculated value of 0.948 (for human males) is virtually identical to the number 0.949 reported by Braselmann et al. for females (4).

A final point to consider is that mFISH typically allows for the detection of asymmetrical exchanges (dicentrics) involving homologous chromosomes, but not their symmetrical counterpart (translocations). In that sense, Eq. (5) “overcorrects” for undetectable exchanges between homologs. If we make the usual assumption that symmetrical and asymmetrical exchanges, as measured by mFISH, occur with approximately equal frequency (23), then half the deviation from unity shown in Eq. (5) no longer applies. Thus, the true frequency of interchanges visible by mFISH – to include dicentrics between homologs (but not translocations) – is given by Eq. (6).

(6) $$F_{\mathrm{mFISH}}=0.948+\left(\frac{1-0.948}{2}\right)=0.974$$

In order to calculate the detection efficiency of WCP, we compare this value to the theoretical frequency of interchanges detectable by three-color FISH, *F_P_* of Eq. (4). As compared to the frequency of interchanges visible by 24-color mFISH, the WGE for such detection by three-color WCP becomes:

(7) $$\begin{array}{l} F_{\mathrm{mFISH}}^{3\;\mathrm{color}}=\left(\frac{2}{0.974}\right)\left[f_{p}\left(1-f_{p}\right)+f_{1}f_{2}+f_{1}f_{4}+f_{2}f_{4}\right]\\ \;=2.053\left[f_{p}\left(1-f_{p}\right)+f_{1}f_{2}+f_{1}f_{4}+f_{2}f_{4}\right]=0.394 \end{array}$$

Thus, by covering 23% of the genome [Eq. (1)], three-color FISH is capable of detecting 39% of the interchanges seen by mFISH. In theory, three-color WCP frequencies can be multiplied by the following correction factor *CF* in order to achieve full 24-color mFISH equivalency.

(8) $$\begin{array}{l} F_{\mathrm{WCP}}\times \mathrm{CF}=F_{\mathrm{mFISH}}\\ \;\mathrm{CF}=\frac{1}{0.394}=2.54 \end{array}$$

This value differs from the CF of 2.9 reported by Braselmann and colleagues, mainly because the three chromosomes we have chosen to analyze (1, 2, and 4) constitute a larger proportion of the genome than the chromosome 1–4–12 triplet used by these authors. Hereafter, the derivation of Eq. (8) will be referred to the CF *derived from first principles*.

### Dose Dependency

As discussed later, correction factors derived from Eq. (8) are of limited value if they display dose dependency. In other words, the transformation of three-color data to WGE is based on the tacit assumption that the two dose–responses can be scaled to match each other (made superimposable)*over a range of doses* through use of a single multiplier, i.e., the constant CF of Eq. (8). It should be intuitively obvious that this is not possible unless certain conditions are met, foremost is that the two dose–responses share the same functional form – a comparison of two linear dose responses would be a trivial example here. However, this alone is insufficient for the general case, as demonstrated for the familiar linear-quadratic formalism of Eq. (9) below. It will be used to describe each of the underlying dose–responses considered in this paper. Let *F*_(D)_ represent the dose-dependent frequency for simple exchanges, as given by the second-order polynomial where α, β ≥ 0.

(9) $$F_{1\left(\text{D}\right)}=\alpha_{1}D+\beta_{1}D^{2}$$

Assume that Eq. (9) represents exchanges as measured by mFISH, and that a similar expression *F*_2(_*_D_*_)_ describes the dose–response as measured by three-color WCP.

(10) $$F_{2\left(D\right)}=\alpha_{2}D+\beta_{2}D^{2}$$

The ratio of Eqs. (9) and (10) defines CF as a function of D, which hereafter is referred to as the *empirically derived* CF.

(11) $$\mathrm{CF}=\frac{F_{1\left(D\right)}}{F_{2\left(D\right)}}=\frac{\alpha_{1}D+\beta_{1}D^{2}}{\alpha_{2}D+\beta_{2}D^{2}}$$

If we let the proportionality constant (*k*) hold the place of CF, then

(12) $$\alpha_{1}D+\beta_{1}D^{2}=k\left(\alpha_{2}D+\beta_{2}D^{2}\right).$$

Equivalently,

(13) $$D\left(\alpha_{1}-k\alpha_{2}\right)+D^{2}\left(\beta_{1}-k\beta_{2}\right)=0.$$

For our purposes, corrections must be applicable over a *range of doses* (interval of D). It follows that if either of the polynomial coefficients in Eq. (13) is non-zero, then the equation is either linear or quadratic, and can therefore have at most two solutions. Therefore Eq. (13) cannot hold over an interval of dose with *k* fixed unless both its coefficients are 0. In this case, the following relationships are satisfied which, as required, are invariant of dose:

(14) $$\begin{array}{l} \alpha_{1}=k\alpha_{2}\\ \beta_{1}=k\beta_{2} \end{array}$$

It then follows that

(15) $$\frac{\alpha_{1}}{\beta_{1}}=\frac{\alpha_{2}}{\beta_{2}}.$$

Thus, formally speaking, the concept of a single CF can be applied to a pair of second-order polynomials only when α/β ratios of the two are equal. In principle, the validity of Eq. (15) can be used to ascertain the appropriateness of using a single CF to convert a three-color WCP data set to full genome equivalency. In practice, we found that designing a statistical test for this purpose to be problematic, largely because AS and TR frequencies are actually subsets mFISH data and, therefore, cannot be considered independent measurements. Thus, whereas testing the identity of ratios in Eq. (15) is conceptually sound (and useful in the discussion that follows) an alternative statistical approach was required to ascertain dose dependency. We used the approach described below, which models the *proportions* of AS interchanges among all interchanges (pAS = AS/mFISH) and true simple (TR) exchanges among AS exchanges (pTR = TR/AS).

### Statistical Analysis

If pAS is constant and independent of radiation dose, then a multiplicative CF can be used to predict the total number of simple exchanges (interchanges) in all chromosomes (mFISH) based on the number of AS exchanges in chromosomes 1, 2, and 4. The same applies to pTR. Alternatively, if either pAS or pTR depend on dose, their dose responses will have slopes statistically different from 0.

We used logistic regression to model the potential dose dependences of pAS and pTR. Using matrix notation, the model structure is summarized as follows, where logit(*x*) = 1/[1 + exp(−*x*)]:

(16) $$\text{logit}\left(o\right)\;=\;\varphi \times \;V\;+\;\varepsilon$$

Here, ***o*** is a vector of outcome variables: predicted pAS and pTR. ***V*** is a vector of radiation doses, φ is a vector of regression coefficients, and ε is a vector of errors.

Three types of radiation dose dependences for pAS and pTR were assessed using this approach:

(a) a dose–response with intercept (the predicted value of pAS or pTR at zero dose) and slope (the predicted rate of change of pAS or pTR per unit dose), with quasi-binomial error distribution;
(b) intercept plus slope with a binomial error distribution; and
(c) intercept only (with slope effectively set to 0 and not counted as an adjustable parameter) having a binomial error distribution. We performed these analyses separately on γ-ray and Fe ion data. Model fitting was performed using R software (version 3.2.0).

According to the binomial distribution, the variance is not an independent parameter, whereas the quasi-binomial option allows the variance to be adjustable (33). Consequently, comparison of binomial and quasi-binomial model fits to the same data (i.e., options a and b), using the X^2^ (Chi-squared) test on residual deviances, provides information on whether or not there is evidence of “overdispersion” in the data – in other words, whether or not the variance of the data is larger than what would be expected from the binomial distribution.

Comparison of the slope plus intercept versus intercept-only models (i.e., options b and c) on the same data provides information on whether or not the data are consistent with being represented by a constant dose-independent term (intercept-only), or if there is evidence for dose dependence (slope). This assessment was performed using the sample-size corrected Akaike information criterion (AICc). AICc is an information theoretic criterion that quantifies relative support from the data for the compared models, taking into account the sample size (number of data points) and the number of adjustable parameters in each model.

Goodness of fit (GOF) was assessed for the models under the assumption that the residual deviance follows the *X*^2^ distribution. The null hypothesis was that the model provides an adequate fit to the data, and small *p*-values were interpreted to mean that the null hypothesis has poor support.

## Results

There are two separate issues to consider when determining how well multiplicative correction factors predict the mFISH dose–responses from three-color data. The overarching first issue is whether such a multiplicative factor *even exits* that can bring three-color data into registry with mFISH data over a range of relevant doses. Obviously, this necessitates that CFs not exhibit dose dependency. That is, assuming a linear-quadratic model for the dose responses that Eq. (15) is not violated.

**Figure 2:**
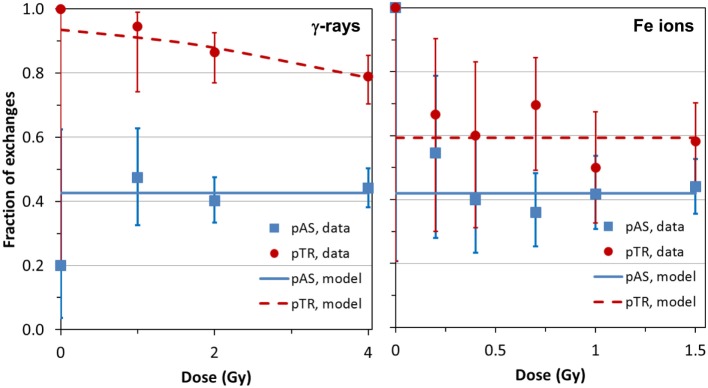
**Data (symbols) and model predictions (curves) for the proportion of apparently simple exchanges among all exchanges (pAS) and for the proportion of true simple exchanges among apparently simple exchanges (pTR)**. Error bars represent 95% confidence intervals (CIs) from the binomial distribution. Details are described in the main text.

The analysis of frequencies of AS and TR, as function of dose for both types of radiation (γ-rays and Fe ions) is shown in Figure 2, which derives from data shown in Table 1. For the densely ionizing Fe ions, there was no evidence for dose dependence for the proportion of AS exchanges among all exchanges (pAS), or for the proportion of TR exchanges among AS ones (pTR). This conclusion was reinforced by the finding that AICc for the intercept-only dose–response model was lower (suggesting higher support from the data), than the AICc for the intercept plus slope model. The best-fit values for pAS and pTR at all doses were 0.420 (95% CI: 0.365, 0.477) and 0.593 (0.505, 0.677), respectively. For sparsely ionizing γ-rays, pAS was also consistent with dose-independence with a best-fit value of 0.426 (0.382, 0.471).

**Table 1:** **Chromosome exchange data and fit parameters**.

IR	Dose (Gy)	No. Cells	^a^AS (3-color)	^b^True (3-color)	^c^mFISH (24-color)	pAS = AS/mFISH	pTR = True/AS
^137^Cs γ-rays	0.0	365	1	1	5	0.200 (0.036, 0.624)^f^	1.000 (0.207, 1.000)
	1.0	238	18	17	38	0.474 (0.325, 0.627)	0.944 (0.742, 0.990)
	2.0	342	74	64	184	0.402 (0.334, 0.474)	0.865 (0.769, 0.925)
	4.0	179	109	86	247	0.441 (0.381, 0.504)	0.789 (0.703, 0.855)
	Σ	1124	202	168	474		

Parameters		^d^α	0.05 ± 0.01	0.06 ± 0.01	0.13 ± 0.05		
		^e^β	0.03 ± 0.00	0.02 ± 0.00	0.06 ± 0.02		
		α/β	2.20 ± 0.01	3.89 ± 0.01	2.27 ± 0.05		
1.1 GeV ^56^Fe Ions	0.0	98	1	1	1	1.000 (0.207, 1.000)	1.000 (0.207, 1.000)
	0.2	191	6	4	11	0.545 (0.280, 0.787)	0.667 (0.300, 0.903)
	0.4	179	10	6	25	0.400 (0.234, 0.593)	0.600 (0.313, 0.832)
	0.7	197	23	16	64	0.359 (0.253, 0.482)	0.696 (0.491, 0.844)
	1.0	189	28	14	67	0.418 (0.307, 0.537)	0.500 (0.326, 0.674)
	1.5	218	55	32	125	0.440 (0.356, 0.528)	0.582 (0.450, 0.703)
	Σ	1072	123	73	293		

Parameters		α	0.14 ± 0.03	0.09 ± 0.03	0.36 ± 0.07		
		β	0.02 ± 0.03	0.00 ± 0.03	0.02 ± 0.06		
		α/β	9.53 ± 0.43	105 ± 82.4	17.6 ± 3.16		

*^a^Apparently simple exchanges; chromosomes 1/2/4; ^b^True simple exchanges; chromosomes 1/2/4; ^c^True simple exchanges; all chromosomes, mFISH; ^d,e^Linear and quadratic coefficients; *Y* = α*D* + β*D*^2^; ^f^Parentheses indicate 95% confidence intervals (CIs)*.

However, the pattern was altogether different for TR exchanges. The γ-ray-induced pTR decreased with dose with a best-fit logistic slope coefficient of −0.3416 (SE: 0.1806, *p* = 0.0585) Gy^−1^. Although the *p*-value for this coefficient was marginally higher than the commonly used significance threshold of 0.05, the intercept and slope model had higher support (by 1.87 AICc units) than the intercept-only model. This favors a response model containing a slope parameter over the intercept-only model having no dose dependence: the strength of evidence for the first model over the second is exp(1.87/2) = 2.54. In other words, although the strength of statistical evidence falls short of being overwhelming, the data suggest that pTR decreases with radiation dose for γ-rays (Figure 2).

From a dose-dependency standpoint, these results show that the response for AS exchange frequencies for gamma rays and ^56^Fe ions are *theoretically* capable of being transformed to match that from mFISH data using a simple multiplicative CF; the same for TR exchanges induced by iron ions. Unfortunately, the same cannot be said for TR exchanges produced by gamma rays, due to the aforementioned dose dependency.

**Figure 3:**
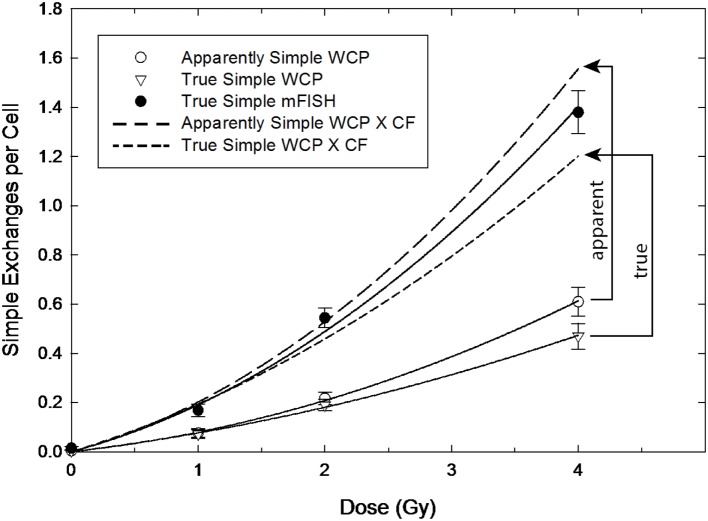
**Gamma ray dose–responses for apparently simple (AS) and true simple (TR) exchanges (open circle and triangle symbols, respectively) as reconstructed from mFISH data**. Arrowed brackets show the predicted dose responses following the application of the CF from Equation (8), which can be compared to the actual whole-genome frequencies for simple exchanges measured by mFISH (solid circles).

**Figure 4:**
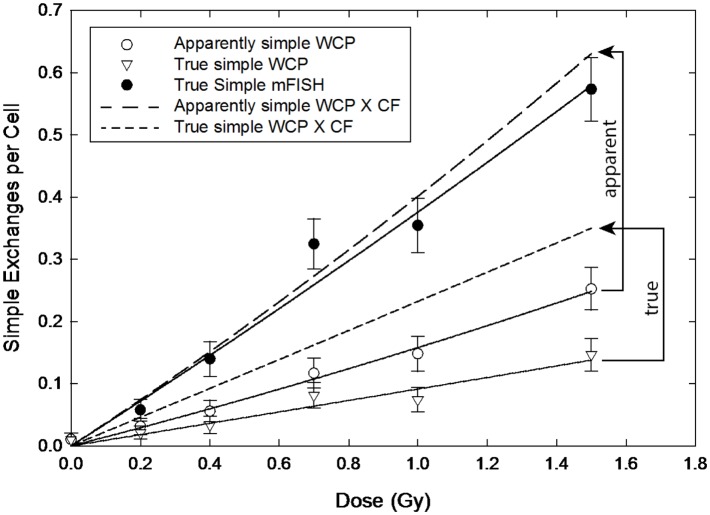
**Dose–responses for apparently simple (AS) and true simple (TR) exchanges (open circle and triangle symbols, respectively) following exposure to ^56^Fe ions**. Arrowed brackets project the dose–responses following the application of the CF from Equation (8). Solid circles are actual whole-genome frequencies for simple exchanges, as measured by mFISH.

Findings concerning dose dependency, however, say nothing about the inherent accuracy of the transformation constant itself, which depends entirely on the assumptions underlying the derivation of Eq. (8). This is the second issue that determines how well a particular CF applied to three-color data predict genome-equivalent frequencies. Figures 3 and 4 are introduced to help visualize the added influence this aspect brings to whole-genome correction. The figures are not intended to imply any sort of rigorous statistical analysis, but to illustrate the overall effect of applying CFs to both AS and TR exchanges. Here, we performed least-squares regression on the data using the linear-quadratic dose–response model [Eqs. (9) and (10)]. The parameters derived from this procedure (Table 1) were used to generate the dose–responses for simple exchanges shown in Figures 3 and 4. The response in lymphocytes exposed to gamma rays is shown in Figure 3. The uppermost solid curve shows a regression to the data (filled circles) for all simple reciprocal exchanges measured by mFISH. These are truly simple exchanges and represent WGE of Eq. (9) having the fitted parameters shown in the table. The open symbols of the figure represent three-color data that was extracted from this dose–response. Open circles show the response for simple exchanges as they would appear to the observer using three-color WCP; see Eq. (10). These are labeled “AS” because (as revealed by mFISH) they are partly comprised of pseudosimple exchanges.

The CF of Eq. (8) was applied to the (extracted) AS data in order to convert them to WGE (i.e., mFISH frequencies). The resultant dose–responses are shown by the two dashed-line curves of the figure. Since pseudosimple exchanges are (by definition) hidden to three-color analysis, CFs can only be applied to AS exchanges during actual three-color painting. The resulting AS to WGE extrapolation (long-dashed curve) is symbolized by the vertical arrowed bracket of the figure labeled “apparent.” As shown in the figure, the extrapolated genome-equivalent dose–response based on three-color WCP systematically *overestimates* the total frequency of simple exchanges measured by mFISH. Nevertheless, as a first approximation for gamma rays, WGE corrections produce results whose accuracy is probably adequate for many purposes, even if only marginally so at higher doses.

A noteworthy aspect of our retrospective analysis is that it also allows the extraction of TR exchanges from the three-color data, as shown by the triangles of the lowermost curve. This represents the dose–response for TR exchanges involving the three painted chromosomes, whose associated fit parameters appear in Table 1.The result of corrections applied to TR *exchanges* is symbolized by the vertical bracket of the figure labeled “true”; it is associated with the dose–response shown by the short-dashed curve. It should be noted that, in this case, extrapolation *underestimates* the frequencies of the mFISH dose–response.

The difference between CFs applied to AS versus TR exchanges is magnified when we consider exposure to 1.1 MeV ^56^Fe ions, as shown in Figure 4. The solid symbols represent full genome equivalence for TR exchanges (mFISH). The open circles and triangles are for AS and TR exchanges, respectively, and represent 3-color data rendered from 24-color mFISH images. As with gamma rays, CFs applied to AS exchanges produced rather good results, although they tended to overestimate the mFISH response. However, the same corrections applied to TRs *grossly underestimated* WGE across the full range of doses examined.

**Figure 5:**
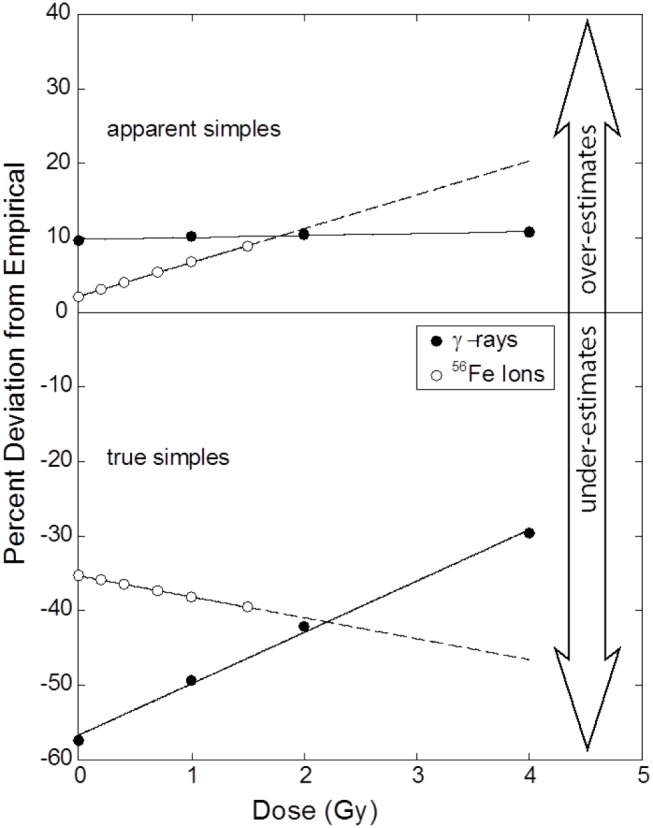
**Percentage errors as a function of dose that result from the application of the CF to AS (upper panel) and TR exchanges (lower panel) for gamma rays and iron ions**. Symbols mark dose points where raw data (Table 1) was collected and whose ordinate values derive from Eq. (17). Errors for AS exchanges induced by gamma rays are invariant of dose. Dose dependency is apparent for the three remaining responses, a result statistically supported for TR exchanges induced by gamma rays. A perfect correction would be represented by a flat dose–response that is centered on 0%. See text for full explanation.

The situation is graphically represented in Figure 5, which compares the results of actual (empirically derived) CFs of Eq. (11) to those derived from first principles of Eq. (8), as expressed by the following Eq. (17).

(17) $$\%\;\mathrm{Deviation}=\left[\frac{\mathrm{CF}\left(\alpha_{3\;\mathrm{color}}+\;\beta_{3\;\mathrm{color}}D\right)}{\alpha_{\mathrm{mFISH}}+\;\beta_{\mathrm{mFISH}}D}-1\right]\times 100$$

The figure shows errors associated with CFs as a function of dose applied to both AS and TR exchanges. The upper portion of the Figure 4 shows deviations as applied to AS exchanges. The errors are positive for both radiation types, meaning that the CF of Eq. (8) *over estimates* the WGE mFISH response. For gamma rays, the deviations are relatively small (~10%) and (as we have shown statistically) are practically invariant of dose. Plotted this way, errors for ^56^Fe ions increase with dose, although as shown in a previous section, this increase could not be validated on the basis of our statistical tests. Rather unexpectedly, errors are practically nil at doses approaching 0, before climbing to about 8% at the highest dose of 1.5 Gy. When extrapolated beyond this dose (extended dashed curve) errors continue to rise in a near-linear fashion, crossing that for gamma rays at ~1.8 Gy, the significance of which is discussed in the following section.

The lower portion of the Figure 5 refers to corrections applied to TR exchanges. Here, extrapolation to WGE badly *underestimates* the true frequency for both types of radiation, as indicated by negative percentage values shown in the figure.

For gamma rays, the (absolute) errors associated with lower doses exceed 50%. Consistent with our statistical analysis, errors decrease sharply with dose, but even at 4 Gy, values are still some 30% lower than those measured by mFISH. Although unsubstantiated by our statistical tests, for ^56^Fe ions there is a seemingly linear increase in relative error with dose, from about 35% at doses approaching 0, to roughly 40% at 1.5 Gy, the highest dose used in these experiments. The response extrapolated to 4 Gy is shown by the dotted line, based on fitted parameters of Table 1.

## Discussion

We feel compelled to point out – after attending to various tedious details specific to our experimental system – that the 2.053 constant appearing in Eq. (7) is practically identical to the 2.05 value originally published by Lucas et al. (3). Actually, we find it amusing that ignoring all such adjustments made subsequent to Eq. (4) leads to a mere 2.5% error by comparison to Eq. (7), which is small compared to the errors shown in Figure 5, and which we imagine would be sufficiently accurate for most experimental purposes.

Clearly, mFISH is capable of providing more cytogenetic information than three-color WCP, including the ability to distinguish TR exchanges from psuedosimples. It is also considerably more demanding of resources and, in many cases, yields data superfluous to the investigator. Presupposing that the two approaches produce quantitatively comparable results, WCP would be the method of choice in instances where less detailed information is an acceptable compromise given its lower expense, rapidity of data acquisition, and ease of analysis.

Regarding the study of whole-genome corrections, there are a couple of points in favor of our retrospective mFISH approach. Unlike earlier studies that sought to establish correlations between mFISH and multi-color data, we establish a proper *correspondence*. In other words, for each and every “three-color cell,” there is a corresponding cell for which 24-color data are obtained. Most WCP studies are vexed by the very prospect of pseudosimple exchanges. For that reason, they are limited to experimental doses and radiation types for which one can reasonably assume the frequency of complex aberrations is minimal, namely low doses of sparsely ionizing radiation. The ability to cull psuedosimples from AS exchanges allows us to analyze full dose–response relationships for TR exchanges, irrespective of dose and radiation quality. Later in this section, we consider the theoretical implications of WGE corrections applied to TR exchanges (TR). But first we discuss the more practical aspects of WGE corrections, which involve their application to AS exchanges.

### Apparently Simple Exchanges

As routinely practiced, WCP to WGE corrections are confined to AS events, simply because WCP is incapable of distinguishing TRs from pseudosimple exchanges. Looking to Figure 3, we find basic agreement with conclusions of Braselmann (4) and others (3, 7, 8) that biophysically based corrections do a reasonably good job of predicting WGE for gamma rays. Applying a multiplicative CF of Eq. (8) to three-color data overestimates mFISH frequencies for simple exchanges, but only by about 10%. Equally important is that the 10% error is essentially constant over the dose interval examined. This is a direct consequence of α/β ratios for mFISH and AS dose–responses being equal, and is reflected in the flat dose–response shown in the leftmost panel (blue-coded data) of Figure 2, and the upper panel of Figure 5 for gamma rays. Thus, reducing the CF of 2.54 in Eq. (8) by 10% would lead to a near perfect match across the full range of doses examined for AS versus mFISH dose–responses shown in Figure 3.

Although not detected with confidence by our statistical methods (Figure 2), α/β ratios for ^56^Fe ions (WCP data versus that of mFISH) are probably not precisely equivalent. This would explain the apparent sloped dose–response shown in the upper panel of Figure 5. If true, then there is no interval of dose over which Eq. (11) is stable enough to be represented by a constant. That being said, the Figure shows that the errors are not large. Across the range of ^56^Fe ion doses studied, they deviate from the empirically derived CF by less than 10%, and actually *diminish* with dose. Said differently, we suspect that Eq. (15) has been violated by some small degree, but as a practical matter, this produces a CF that would be deemed acceptable for many purposes. At first glance, these results seem counterintuitive, since high LET radiations are known to produce copious quantities of complex aberrations that are cryptically embedded in the AS data as pseudosimples. However, they are entirely consistent with the interpretation that, to a first approximation, all effects from high LET radiation are “intra-track.” Consequently, there would be the same fixed fraction of aberrations (of any kind, including complex exchanges) per unit dose of Fe ions.

Chromosome aberrations are a viable surrogate endpoint for mutations and cancer, and have long been the *de facto* “gold standard” for biodosimetry (34). WCP to WGE corrections, therefore, have implications for radiation protection, where concerns over the biological effects of very low doses are paramount. At issue is the concept of relative biological effectiveness (RBE), which for the present work, involves a comparison between the effects of gamma rays and heavy ions. Here, WCP finds a place because of cost and sample throughput considerations related to the need to score many cells.

As shown graphically in Figure 5, it is debatable whether a common CF can be assigned to *both types* of radiation that is valid over a range of doses. It was previously mentioned that the upper portion of the Figure shows that AS errors for both radiations intersect at about 2 Gy. A common CF is valid for both radiations only at this dose, which is far too large to be of practical use as regards issues of radiation risk. At more relevant lower doses, the two curves diverge sharply. This significantly complicates RBE calculations, which are made on the basis of the ratio of doses for a given isoeffect. For RBEs other than unity, the isoeffective doses will differ, meaning that separate CFs would need to be applied to each type of radiation. Moreover, in the case of 1.1 GeV ^56^Fe ions used in these studies, CFs will also change depending on the chosen level of isoeffect. That said, and as a practical matter, Figure 4 indicates these errors are capable of altering RBE values for ^56^Fe ions by about 8% at very low doses. Whereas these errors do not seem particularly large in an experimental setting, in the context of RBE-related radiation protection issues, they probably should not be ignored.

### True Simple Exchanges

The remaining discussion focuses on TR exchanges. Recall that the presence of complex aberrations – in this case, taking the form of pseudosimple exchanges in AS data – is specifically ignored during the derivation of Eq. (8), and only simple pairwise exchanges are considered. So, in theory, one would imagine that CFs applied to TR exchanges would produce better results than CFs applied to AS exchanges. As we have seen, the opposite is true. Errors of mFISH predictions based on TR show a pronounced dose dependency for γ-rays (Figure 2), and underestimates of WGE for both radiation types occur (Figures 3 and 4). From a predictive standpoint, the errors for ^56^Fe ions are severe enough to render such extrapolation practically useless. Ironically then, after culling pseudosimples from the data – thus satisfying a principle assumption underlying the derivation of Eq. (8) – the resulting predictions were much poorer than if CFs were applied to AS exchanges. Said differently, our data show that the “contaminating” influence of pseudo simple exchanges actually serves to *improve* the predictive ability of WGE corrections. So, to the extent that WGE corrections are considered sufficiently accurate, they owe this accuracy to the very presence of complex aberrations! While this result may be reassuring from a practical standpoint, from theoretical perspective it is disconcerting, because it implies either that the basic approach underpinning WCP-to-WGE conversion is fundamentally flawed, or that a violation of some primary assumption has taken place.

### The Discrepancy for True Simples

The derivation of Eq. (8) makes the assumption of *random* pairwise interactions between primary radiogenic breaks leading to interchanges. In that case, variance/mean ratios of unity should result, consistent with the expectations of a Poisson distribution (35, 36). The total counts of chromosomal exchanges in the genome (mFISH), as well as subsets of these data – AS and TR exchanges – showed no clear evidence of overdispersion. For both γ-rays and Fe ions, the dispersion parameter in quasi-binomial fits was always in the range of 0.58–1.19, close to unity. The X^2^ test for residual deviances, which compares the fits of dose–response models with binomial and quasi-binomial errors, produced *p*-values of 0.43–0.88, also suggesting no overdispersion. These results are largely consistent with the analysis of the raw data to which the U-test (36) was also applied to check σ^2^/Y ratios for overdispersion (data not shown). By applying this latter criterion to the data for gamma rays, we found no evidence that the distribution of TR or AS exchanges deviated from that of the Poisson. For the high LET ^56^Fe ions, significant over dispersion was detected by the U-test, but only for one of the five doses examined. From this, we conclude that systematic deviation from randomness in the distribution of exchanges per cell is not the principle cause for the failure of Eq. (8) to predict the outcomes of TR exchanges measured by mFISH.

We think a more likely explanation for the large discrepancy involves the remaining fundamental assumption attached to the derivation of Eq. (8), namely that the probability of an exchange between two chromosomes is a product of their proportional genomic content. [We hasten to make a minor point here that, strictly speaking, it is probably more accurate to consider the length of interphase chromosome arms, or chromatin fibers (the chromonema) of individual chromosomes in such interactions (37), rather than gross DNA content, although the two parameters are sufficiently related (38) that they can probably be used interchangeably in the present context.]

During interphase, it is now well established that chromosomes occupy rather distinct globular domains (39–41), which, it is reasonable to assume, severely limit the opportunity for the interaction of radiogenic breaks between different chromosomes. Consequently, models have been developed that consider interchanges constrained to boundary regions where two chromosomes abut (42), in which case interchanges would be proportional to the product of domain surface areas. For chromosome domains of spherical shape, exchange frequencies would, therefore, be proportional to [DNA content]^2/3^ (43). For spherical domains of radii *r*, the ratio of volume to surface area varies as *r*/3. Consequently, by comparison to models based on volume, predictions based on DNA content tend to systematically overestimate exchange frequencies involving larger chromosomes (9, 44, 45). Unfortunately, this leads to a further lowering of predicted frequencies for exchanges involving the large-sized chromosomes examined here – the opposite of what is needed to bring three-color data in line with that of mFISH for TRs (Figure 5; lower panel). The problem is further exacerbated when one considers that chromosome domains are not actually spherical, but globular instead, because for any irregular volume the surface-to-volume ratio is larger than that of a sphere, or for that matter, any platonic solid.

One should appreciate that simple pairwise exchanges do not form in a vacuum, meaning their formation is always in potential competition with the formation of complex exchanges. Said another way, simple and complex exchanges often compete for the same radiogenic breaks. Consider a constellation of four such *proximate* breaks, defined as breaks that – by virtue of being close in time and space – are capable of freely interacting (rejoining) with one another. An obvious rejoining possibility is that the four breaks rejoin in such a way as to give two simple exchanges. But, should any other misrejoining possibility occur, a complex exchange will result, simultaneously negating the possibility of simple pairwise exchange. Crudely put, the formation of complex exchanges can be envisioned to “steal away” breaks that would otherwise be destined to become involved in forming simple exchanges (46). In this sense, complex exchanges form *at the expense* of simple exchanges, a process that is bound to be dose dependent, since it is strongly influenced by lesion density. To our knowledge, no one has formally modeled this scenario, but it most assuredly would have the overall effect of depressing the expected yields of simple exchanges.

Other explanations for our results involve the higher order organization of the mammalian cell genome (i.e., beyond that of the 30 nm chromatin fiber). Since this remains as one of the least understood aspects of cell biology, a fair amount of speculation is unavoidable. Until now, we have assumed random interaction between radiogenic breaks – either those contained within interphase chromosome domains, or those associated with their surface areas. In fact, the radial distribution of chromosomes in the nucleus is often not random, and differs among cell type and stage of cellular differentiation (47). There is some evidence that larger human chromosomes tend to be located near the periphery of the nucleus. If true, then larger chromosomes would share a proportion of their surface area with the outside nuclear boundary – regions that presumably would be unavailable for interaction with that of more interior domains (48, 49). Such is the case for chromosome 1, at least in fibroblasts (50, 51), although there is also evidence to the contrary for lymphocytes (52, 53). Such a relationship (in principle) would necessitate a higher correction factor be applied exchanges involving the large-sized chromosomes used in this study, which would have the effect of reducing CF errors shown in Figure 5 for TRs.

The assumption of random breakage also implies a more-or-less uniform breakage per unit length of DNA or chromatin. While this is probably true for the initial radiogenic lesions (i.e., DNA double-strand breaks), there is evidence that exchanges themselves occur preferentially in G-light bands (54, 55) or at the interface between light and dark bands (56). These regions, particularly T-bands (a subset of G-light bands) have a much higher than average gene density (57). As long as these “sensitive” regions are randomly distributed among various chromosomes, this should not materially affect the underlying assumptions relating to Eq. (8). However, certain chromosomes are known to be gene-rich, on average, a case in point being chromosome 19 (41) which, perhaps not by coincidence, is thought to occupy an interior position within the nucleus (58). By this argument, gene-rich chromosomes may be subject to increased exchange involvement compared to chromosomes with lower gene density. Additionally, if one equates gene density with transcriptional activity, then the DNA of such regions would presumably have a more “open” or diffuse structure (59, 60), and consequently be less dense in terms DNA content/surface area. In this way, increased transcription associated with gene-rich regions (or whole chromosomes) may have the secondary effect of lowering the physical density of DNA per unit volume. The opposite would be true of more gene-poor chromosomes. With respect to interactions based on surface area, larger chromosomes would then require larger CFs to compensate, again mitigating CF discrepancies shown in Figure 5. These ideas represent little more than a speculative attempt to explain our findings. Whether or not they help to resolve the thorny issue of dose dependency of CFs associated with violation of Eq. (15) remains to be seen.

## Conclusion

The good news – at least from a utilitarian perspective – is that for the purpose of converting WCP data to WGE, CFs work reasonably well across the range of doses to which they are usually applied. The less welcome news is the large discrepancy between WGE-corrected TR exchange frequencies compared to those detected by mFISH, which implies problems with the biophysical underpinnings upon which the derivation CFs rely. We imagine that the fundamental assumptions underlying Eq. (8) are overly simplistic, failing to account for structural features of chromatin, and its dynamic interactions within the interphase nucleus. Sophisticated methods are being applied to this area of study that, in principle, can provide the investigator a “snapshot” into physical relationships that exist between interphase chromosomes (61, 62), but these fall short in addressing any potential time-dependent dynamic interactions. We have known, for the better part of a century, that initial radiogenic breaks in chromosomes need to be both spatially and temporally close for exchanges to occur (63). And yet, there are almost certainly aspects of this relationship that we do not fully understand. A phrase used often by the preeminent cytogeneticist J.R.K Savage seems an appropriate closing note: “Everything is more complex than it appears at first sight.”

## Author Contributions

BL conducted the experiments and helped analyze the data. IS provided critical statistical support and helped edit the manuscript. MC conceived of the experiment, provided financial support, and wrote most of the paper.

## Conflict of Interest Statement

The authors declare that the research was conducted in the absence of any commercial or financial relationships that could be construed as a potential conflict of interest.
